# Circumpapillary Retinal Nerve Fiber Layer OCT Imaging in a Parkinson’s Disease Cohort—A Multidisciplinary Approach in a Clinical Research Hospital

**DOI:** 10.3390/jpm12010080

**Published:** 2022-01-09

**Authors:** Vlad-Ioan Suciu, Corina-Iuliana Suciu, Simona-Delia Nicoară, Lăcrămioara Perju-Dumbravă

**Affiliations:** 1 Department of Neuroscience, “Iuliu Haţieganu” University of Medicine and Pharmacy, 400012 Cluj-Napoca, Romania; lperjud@gmail.com; 2 Department of Ophthalmology, “Iuliu Haţieganu” University of Medicine and Pharmacy, 400012 Cluj-Napoca, Romania; scorinamail2019@gmail.com (C.-I.S.); simonanicoara1@gmail.com (S.-D.N.)

**Keywords:** multidisciplinary approach, non-invasive diagnostic tool, optical coherence tomography, parkinson’s disease, personalized medicine

## Abstract

(1) Background: The purpose of this paper is to report the data of the first study in a Clinical Research Hospital, in the Transylvania region, focusing on the Spectral Domain Optical Coherence Tomography (SD-OCT) measurements in the early stages of Parkinson’s disease (PD), and to compare the results with age-matched healthy controls. (2) Methods: This study assessed the circumpapillary retinal nerve fiber layer (cpRNFL) SD-OCT measurements (Heidelberg Spectralis, Heidelberg Engineering, Germany) of two study groups: patients suffering from PD (Hoehn−Yahr stages 1–3) and healthy controls. Secondary objectives were to investigate the reported visual symptoms by evaluating the color vision, contrast sensitivity, and the central visual defects for macular disease using standardized charts. Subjects with prior history of ophthalmologic diseases, advanced stages of PD (Hoehn−Yahr stages 4–5), or with psychiatric conditions were not included in this study. The same team of neurologists and ophthalmologists evaluated all individuals in order to have comparable data and to eliminate inter-examiner differences. All subjects were recruited from the same Clinical Research Hospital in the Transylvania region, Romania. (3) Results: 72% of the PD patients (*n* = 17) in this study reported visual symptoms. In respect to the ophthalmologic chart evaluation for PD patients, the most frequent disturbances were identified in the Ishihara color perception testing (33%). The regression analysis showed significant results for the Ishihara testing in relation to the cpRNFL thinning in the temporal retinal sectors for both eyes. cpRNFL thinning was predominantly contralateral to the parkinsonism (*p* = 0.001). The temporal and global values of the cpRNFL were significantly lower in all PD patients < 70 years old, compared to the age-matched healthy controls. (4) Conclusions: Specific patterns of cpRNFL thinning were found in the PD subjects younger than 70 years. A multidisciplinary approach is essential for a complete evaluation of PD patients.

**Highlights**

- More than two thirds of the PD patients reported at least one visual symptom
- 33% of the PD patients had color vision disturbances
- cpRNFL thinning was predominantly contralateral to the parkinsonism (*p* = 0.001)
- Significant cpRNFL thinning was found in the temporal and global values of all PD patients younger than 70 years
- There was no correlation between the Hoehn-Yahr stage and the global cpRNFL value (µm)

## 1. Introduction

More than 200 years have passed since James Parkinson first described in detail “the shaking palsy”, known today as idiopathic Parkinson’s disease (PD). Researchers and clinical practitioners continuously discover and study this complex neurodegenerative pathology. PD has gone through many epochs of both diagnosis and management, being, nowadays, mostly clinically diagnosed and unsatisfactorily treated with dopamine substitutes. We know today that PD is characterized by a combination of both, the well-known motor and the non-motor symptoms, including visual function changes [1,2,3].

Optical coherence tomography (OCT) is a non-invasive, easy-to-perform technique, which uses the infrared light to scan the retinal structure. Some authors compare OCT (near histological detail: 7 µm axial and 14 µm transverse resolution) with ultrasonography (150 µm axial resolution of a conventional B-mode image), because of the similar principles involved in the scanning process. The value of OCT is well established in ophthalmology, but it has recently grown to provide neurologists with important information regarding the retinal changes in different neurological diseases (e.g., multiple sclerosis and Alzheimer’s disease). Because the retina is part of the central nervous system, it is important to have a tool that can analyze it in vivo, non-invasively [4,5,6,7,8,9].

It is known that the retinal dopaminergic cells send axonal projections to the inner and outer plexiform layers, synapsing with the horizontal cells. If these cells are affected in PD, then thinning of the inner retinal layers could be found by OCT examination. [5,6,10,11,12]. From this supposition, previous papers demonstrated retinal thinning in different locations: temporal-inferior quadrant [13,14], nasal-inferior quadrant [10], and temporal quadrant [15,16]. Visser and Yu support the theory that the temporal retinal quadrant contains the papillo-macular fibers, which could be more susceptible to neurodegeneration. The mitochondrial dysfunction in PD could lead to the axonal loss of the papillo-macular bundle [15,17,18].

Regarding the lateralization of cpRNFL thinning and the predominantly affected body side in PD, other authors have found that retinal thinning is contralateral to the parkinsonism [17,19,20]. Adams et al. brought up the theory of a link between the basal ganglia and the retina [21].

Most of the patients included in our study come from Cluj County, which is one of the most populated areas in Transylvania [22,23]. In 2011, according to the National Institute of Statistics in Romania, the total population in Cluj was 691.106, the majority being Romanian (80%) and Hungarian (15.90%) inhabitants, having a rich genetic diversity [22]. The European population has genetic variations and specific patterns for each geographic region [24]. In the process of finding similarities or differences in various geographic regions of the world, we could get closer towards patient tailored medicine for the future.

The purpose of our research is to investigate and compare the circumpapillary retinal nerve fiber layer (cpRNFL) measurements in PD patients with age-matched healthy controls. By doing so, we intend to evaluate the SD-OCT measurements in the early stages of PD. Secondary outcomes are to investigate PD-related visual symptoms and to characterize the study population.

A distinctive feature of our research is the analysis of each retinal sector (*n* = 6) in the individual age groups, and the comparison between the two study samples (PD and controls). We also studied the lateralization of the cpRNFL in respect to the parkinsonism and evaluated the relation between cpRNFL values and disease severity. To our knowledge, this is the first study of this kind that has been done in a Clinical Research Hospital, in the Transylvania region, Romania. By studying the OCT measurements in this region, we can compare the data of our research to previous studies and point out the similarities or differences.

## 2. Materials and Methods

This observational, prospective study evaluated six retinal sectors in 30-patient eyes (*n* = 17 PD patients) with Parkinson’s disease in the early stages of evolution, and 23 eyes of age-matched healthy controls (*n* = 17 healthy patients), all of which were Caucasian individuals. For each PD patient in this study, we enrolled an age-matched healthy control (HC). In addition, age stratification was done for both study groups, in order to control this potential confounder. The PD patients were recruited from the Neurology Clinic I of the Emergency County Hospital Cluj-Napoca, while the HC were recruited from the Ophthalmology Clinic of the same hospital. Healthy individuals were considered those who did not suffer from any chronic neurologic or ophthalmologic disease.

All PD patients considered in this study were examined by the same neurologist and ophthalmologist throughout the study, and signed an informed consent before enrolling. The study protocol (protocol number 487/21.11.2019) adheres to the Declaration of Helsinki, and was approved by the review boards of the University of Medicine and Pharmacy “Iuliu Haţieganu” Cluj-Napoca, Romania, and the Emergency County Hospital Cluj-Napoca, Romania. All necessary precautions were taken to preserve the confidentiality of the patients’ data.

The inclusion criteria were as follows: patients > 18 years, suffering from PD (Hoehn−Yahr stages 1–3), who agreed to participate in the study. Patients with a medical history of a severe ocular disease (glaucoma, optic neuropathy, cataract, and advanced diabetic retinopathy), which could influence the results, and those with advanced stages of PD and related complications, were excluded. All patients were receiving optimal dopaminergic medication at the time of study enrollment. Examination took place in the “on-therapy” period in order to avoid examination difficulties. All subjects had normal intraocular pressure values measured by aplanotonometry.

All enrolled individuals (PD patients and HC) underwent a complete neurologic and ophthalmologic examination, including visual acuity testing, visual field exam, ophthalmoscopic examination, biomicroscopy, and measurement of the intraocular pressure. The color vision testing was done, using the Ishihara charts, for both eyes separately. The central visual defects for macular disease were tested with the Amsler grid (black grid on a white background enclosing 400 smaller 5 mm squares). The contrast sensitivity was tested with the Pelli-Robson letter chart. A questionnaire was used for reporting the visual symptoms.

**Figure 1:**
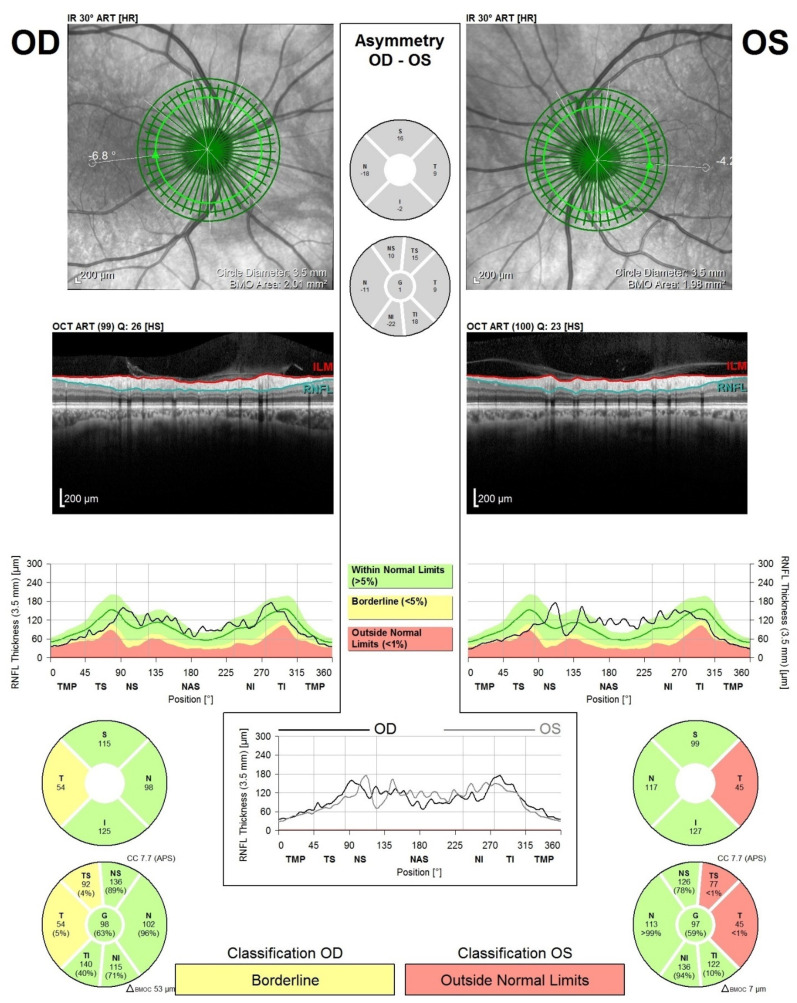
Heidelberg Spectralis OCT acquisition of a cpRNFL scan in a PD patient. The cpRNFL scan was divided into six retinal sectors: temporal, temporal-superior (TS), nasal-superior (NS), nasal (N), nasal-inferior (NI), and temporal-inferior (TI). OD-oculus dexter; OS-oculus sinister. In this particular case, the cpRNFL of the left eye was affected in the temporal and temporal-superior sectors.

Every acquired OCT image was evaluated by the same ophthalmologist, and low-quality measurements or artefacts of the raw OCT data were excluded from analysis. Fifteen eyes were excluded (*n* = 4 PD and *n* = 11 HC) from the OCT analysis due to various causes (low compliance, ocular structure changes, newly discovered opacities, and incomplete data). The Spectralis SD-OCT device (The Heidelberg Engineering Company, Heidelberg, Germany) was used to examine all subjects in order to have comparative data. The SD-OCT device had the Eyetracking module enabled, in order to prevent motion artefacts due to tremor or eye movements. The cpRNFL thickness was measured in six retinal sectors: temporal, temporal-superior (TS), nasal-superior (NS), nasal (N), nasal-inferior (NI), temporal-inferior (TI), and a global value was automatically provided by the machine’s software (see Figure 1).

The statistical analysis and comparison of the cpRNFL measurements in PD patients with age-matched healthy controls were done using Microsoft Office Professional Plus 2016 (MSO 64-bit version) and CDC Epi Info version 7.2.4.0 software. For the data analysis, we used tables and graphs of the most significant results, and descriptive analyses were represented as mean ± standard deviation (SD). ANOVA and Student’s *t*-test for independent samples were used to compare the mean cpRNFL thickness of PD patients with age-matched controls. Regression models for multiple comparisons were also used. In order to increase the statistical accuracy, we stratified each subject into age groups. For the statistical analysis, significant results were considered for a *p* value < 0.05, which were corrected by the Bonferroni method for multiple comparisons.

## 3. Results

The preliminary results of this ongoing study included 30-patient eyes with PD in the early stages of evolution and 23 age-matched healthy control eyes.

### 3.1. Demographical Characteristics of the Study Population

The mean age of the PD patients was 68 ± 8.92 years, while that of the controls was 57.12 ± 10.95 years. Most PD subjects were in the 60–69 year (44%) and 70–79 year (39%) age groups. The gender distribution of the PD subjects showed an asymmetry, favoring men (78%) versus women (22%).

Most PD patients come from urban areas of Transylvania, Romania (94%), and are retired from their professional activity, according to the legal age (89%). Furthermore, 61% of the PD subjects report no professional toxic exposure in the past, while 89% are non-smokers.

### 3.2. Clinical Characteristics of the PD Sample

The most frequently encountered PD subtype was the tremor predominant type (66.5%), versus the akinetic-rigid predominant type (27.5%) and the equivalent type (6%) (see Table 1).

Moreover, 44% of PD patients had a disease evolution of ≤ 5 years (mean 3.17 ± 1.03 years), while the longer disease evolution categories had a similar distribution (17–22%). The analysis of the PD subjects according to the MMSE scores revealed a distribution in favor of the MMSE scores of 25–29 points (50%) and 30 points (38.8%). The Hoehn−Yahr grading scale showed an approximate similar distribution between stages 2–3.

**Figure 2:**
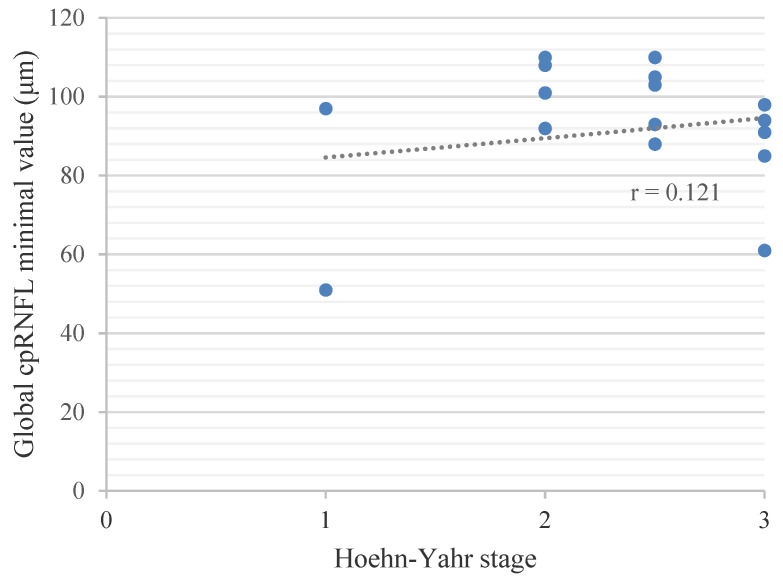
Pearson’s Correlation between the Hoehn−Yahr stage and the Global cpRNFL Value.

There was no correlation between the Hoehn-Yahr stage and the global cpRNFL value (µm) with *r* = 0.121 in the PD sample. (see Figure 2)

The ophthalmologic chart evaluations showed the highest percentage of disturbances in the PD sample for the color perception tests (33%), compared to the Amsler grid distortions (11%) and the modified contrast sensitivity (22%).

### 3.3. Lateralization of the Motor Signs and SD-OCT Retinal Imaging

In the PD sample, the most motor-affected body side was the right side (65%), while the most predominant retinal thinning was observed in the left eye (71%). The analysis of the lateralization of the parkinsonism in relation to cpRNFL thinning revealed that the predominant retinal thinning measured by OCT was contralateral to the parkinsonism (*p* = 0.001).

Lateralization was analyzed for each PD individual separately (see Table 2). Moreover, the correlation coefficient was −0.376 (inverse) between the parkinsonism and the predominant retinal layer thinning in the PD sample with the individual analysis (*p* = 0.002; F 10.6 > F critical 4.1).

In Table 3, we sorted each subject, both the PD patients and controls, into age groups, accordingly, for an accurate comparison of the mean cpRNFL measurements. ANOVA and Student’s *t*-test for independent samples were used to compare the mean cpRNFL thickness of PD patients with age-matched controls, and revealed statistically significant results in the temporal, nasal-inferior (NI), and global value of all age groups < 70 years old. Each age group displayed specific patterns for thinning. In the 40–49 year old age group, the temporal, nasal-superior (NS), nasal-inferior (NI), and global value were significantly thinner in both tests (ANOVA and *t*-test). In the 50–59 year old age group, the temporal, temporal-superior (TS), NI, and global value were significantly thinner, while in the 60–69 year old age group, the temporal, TS, NI, temporal-inferior (TI), and global value were thinner.

Highly significant results stood out for the global value in the 50–59 year old age group (ANOVA *p* = 0.001), but also for the TI value in the 60–69 year old age group (ANOVA *p* = 0.0002).

**Figure 3:**
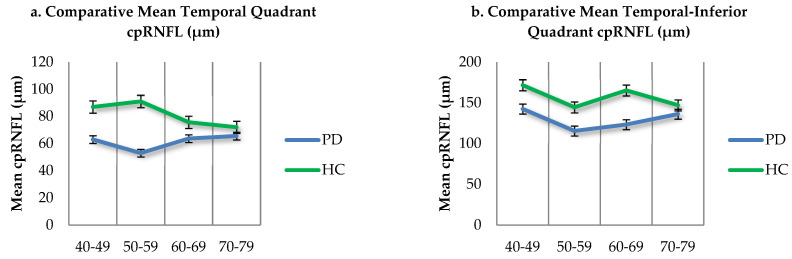

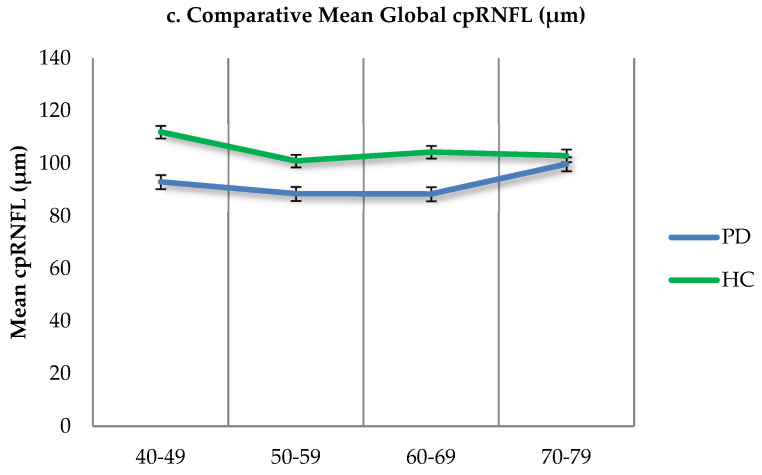
(**a**–**c**). Comparative mean cpRNFL values in PD patients and age matched controls. Standard error—black bars.

The graphical representation for the comparison of the mean cpRNFL values (µm) of the temporal, TI quadrants, and global values in the PD sample (blue) and controls (green), according to each age group, is visualized in Figure 3.

The regression analysis for multiple comparisons showed significant *p* values for the Ishihara testing in relation to the cpRNFL thinning in the right eyes for the temporal quadrant (*p* = 0.014) and global value (*p* = 0.004), and in the left eyes for the temporal quadrant (*p* = 0.047). In addition, the presence of the non-motor symptoms showed significant results for the right eye global value (*p* = 0.040) and the left eye temporal quadrant (*p* = 0.011) and global value (*p* = 0.048; see Table 4).

## 4. Discussion

### 4.1. Visual Function Changes in PD

Idiopathic PD is considered to be a common neurodegenerative disorder affecting a wide range of neurons in regions such as the nigral substance, locus ceruleus, dorsal nucleus of the vagus nerve, the reticular substance of the mesencephalon, the sympathetic ganglia, the olfactory nuclei, and the retina. Based on a hysto-pathological analysis, Braak and colleagues debated that the pathologic process begins in the vagus nuclei and olfactory bulb [1,25].

Idiopathic PD has a complex clinical picture consisting of both motor and non-motor features [1,3].

The clinical onset (with the classic motor symptoms) of PD is between the ages of 45 and 70 years old, but many authors believe that the pathological process begins as early as 10 years before the first clinically evident signs and symptoms. This observation might be an important milestone in trying to find a specific and sensitive biomarker to diagnose PD reliably in earlier stages of evolution [1,10,11,25,26].

In the category of non-motor features, visual function changes were found to be present in 78% of the parkinsonian patients, according to Sauerbier and colleagues in 2013 [11]. Despite the impact they have on the patients’ quality of life (QoL), visual symptoms are underdiagnosed [11]. The present study confirmed this observation by revealing that 72% of the PD patients reported at least one visual symptom. The reported visual function changes in our study were blurry vision, dry eyes, floaters, and difficulty in reading a text (various and intricate causes). Furthermore, in our study, the ophthalmologic chart evaluations revealed color perception anomalies (33%), Amsler grid distortions (11%), and modified contrast sensitivity (22%) in the PD subjects.

In 2000, Pieri and colleagues revealed that PD patients often complained of blurry vision, which they thought to be influenced by disturbances in color discrimination and contrast sensitivity. It is believed that the perifoveal dopaminergic cells are reduced in PD. Some authors have stated that the visual function may be influenced by medication (Levodopa), although Pieri and colleagues did not find any correlation in their study [27]. In our study, all patients received optimal dopaminergic medication at the time of study enrollment, and examination took place in the “on” therapy period. In addition to this, we did not analyze the subjects off therapy, because our study was purely observational, and we also took into account ethical considerations.

All these visual disturbances have complex pathogenetic mechanisms and many have insufficient explanations. Some authors believe that dopamine has an important role in retinal function. D1 and D2 receptors have been found in the retina. These dopamine receptors are implicated in the transition from nocturnal to diurnal vision, but also in pre-processing images, increasing the contrast sensitivity, color discrimination, and others [11].

### 4.2. Retinal Thinning in PD

**Figure 4:**
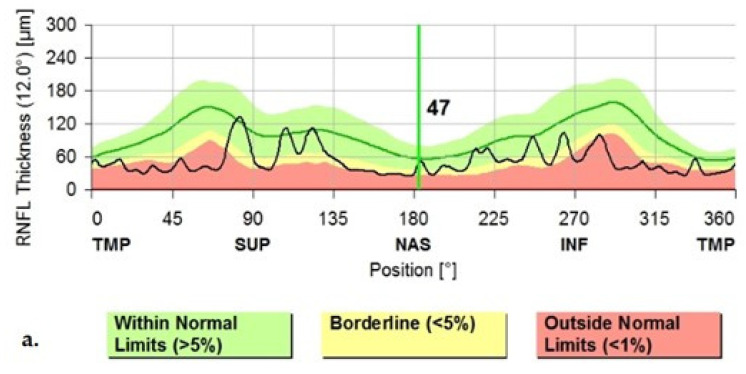

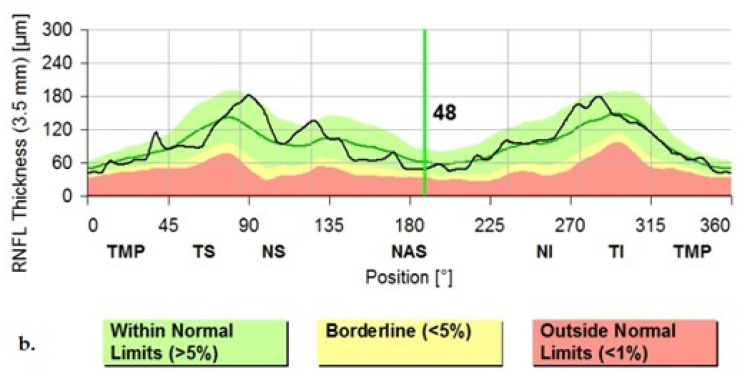
(**a**) The Graphical Analysis of the cpRNFL values in a PD patient (note the black line); (**b**) The Graphical Aspect of the cpRNFL values in a normal subject (Images from our study). TMP—temporal; TS—superior-temporal; NS—nasal-superior; NAS—nasal; NI—inferior-nasal; TI—inferior-temporal.

The main dopaminergic neurons in the retina are considered to be the amacrine and interplexiform cells. These neurons send axonal projections into the internal and external plexiform layers of the retina towards the horizontal cells. Thus, dopaminergic cell loss in the retina would generate a thinning of the inner retinal layers (see Figure 4), which could be detected by OCT [5,6,10,11,12].

In our study, the cpRNFL was thinner, with statistically significant results in the temporal, NI, and global value of all age groups < 70 years old. Each age group displayed specific patterns for thinning. In the 40–49 year old age group, the temporal, NS, NI, and global values were significantly thinner in both tests (ANOVA and *t*-test). In the 50–59 year old age group, the temporal, TS, NI, and global values were significantly thinner, while in the 60–69 year old age group, the temporal, TS, NI, TI, and global value were thinner.

Moreover, highly significant results stood out for the global value in the 50–59 year old age group (ANOVA *p* = 0.001), but also for the TI value in the 60–69 year old age group (ANOVA *p* = 0.0002).

Taking into account the Bonferroni method for multiple comparisons, after correction, the temporal quadrant and global values were significantly thinner in the PD sample for all patient groups < 70 years old, while the NI quadrants were significantly thinner only for the 50–59 year old and 60–69 year old groups. The NS quadrant was significantly thinner only for the 40–49 year old group, while the TI quadrant only for the 60–69 year old group.

The statistical significance for subjects < 70 years of age could mean that the value for the cpRNFL SD-OCT measurements was maximal in the early stages of PD and in younger patients. Age related changes of the retina could probably be the cause of the lack of significant differences in patients older than 70 years, although more studies are needed to confirm this supposition. Our study showed no significant differences between the cpRNFL measurements in the PD sample and the HC in the age group over 70 years. Another important aspect of our work is the fact that we found specific retinal quadrant changes in different age groups. We could assume that each age group < 70 years of age has specific retinal quadrant changes in the early stages of PD (Hoehn−Yahr stages 1–3).

In 2004, Inzelberg and colleagues studied the thickness of the peripapillary retinal nerve fiber layer in PD by using an OCT device, and demonstrated that the temporal-inferior (TI) sector was thinner in PD compared to the healthy controls [13]. They also argued that the visual disturbances related to PD developed in parallel with the motor disturbances and could also fluctuate. Dopamine seems to influence also other neurotransmitters in the retina, such as glutamate, GABA, and glycine, meaning that dopamine depletion can generate a long-term and complex influence on the retinal circuitry [13].

In 2014, a meta-analysis conducted by Ji-guo Yu and colleagues on 13 studies comparing the RNFL thickness by OCT in PD with healthy controls, revealed a significant thinning mostly in the temporal retinal quadrant in PD patients. They argued that the temporal fibers, involving the papillomacular bundle, are more susceptible to neuro-degenerative disease processes. It is, however, relevant to specify that these studies were based on different OCT devices [16]. Visser et al. (2018) confirmed that PD patients had lower retinal nerve fiber layer values in the temporal quadrant [15].

In 2014, Schneider and colleagues presented the results of their study on parkinsonian monkeys, who were evaluated with OCT in vivo. They demonstrated the thinning of the RNFL in the nasal-inferior (NI) quadrants of the retina in the predominant akinetic-rigid forms of parkinsonian monkeys. The macular volume and foveal thickness were also reduced [28].

The NI quadrants were found to be thinner in the akinetic-rigid type of PD compared to the tremor dominant type, according to Rohani et al., based on his study of 27 PD patients [10].

Another study conducted by Jeeyun Ahn et al. in 2018 showed, by examining PD patients with OCT and PET scans, that the retinal thinning in the TI quadrants revealed by OCT was correlated well with the dopaminergic loss in the nigral substance revealed by the PET scans. They emphasized the importance of the OCT in evaluating PD patients in the early stages of the disease [14].

In 2015, Slotnick et al. studied the foveal thickness by OCT in 72 PD patients and concluded that the superior-inferior foveal slope was mostly thinned in these patients [29].

**Figure 5:**
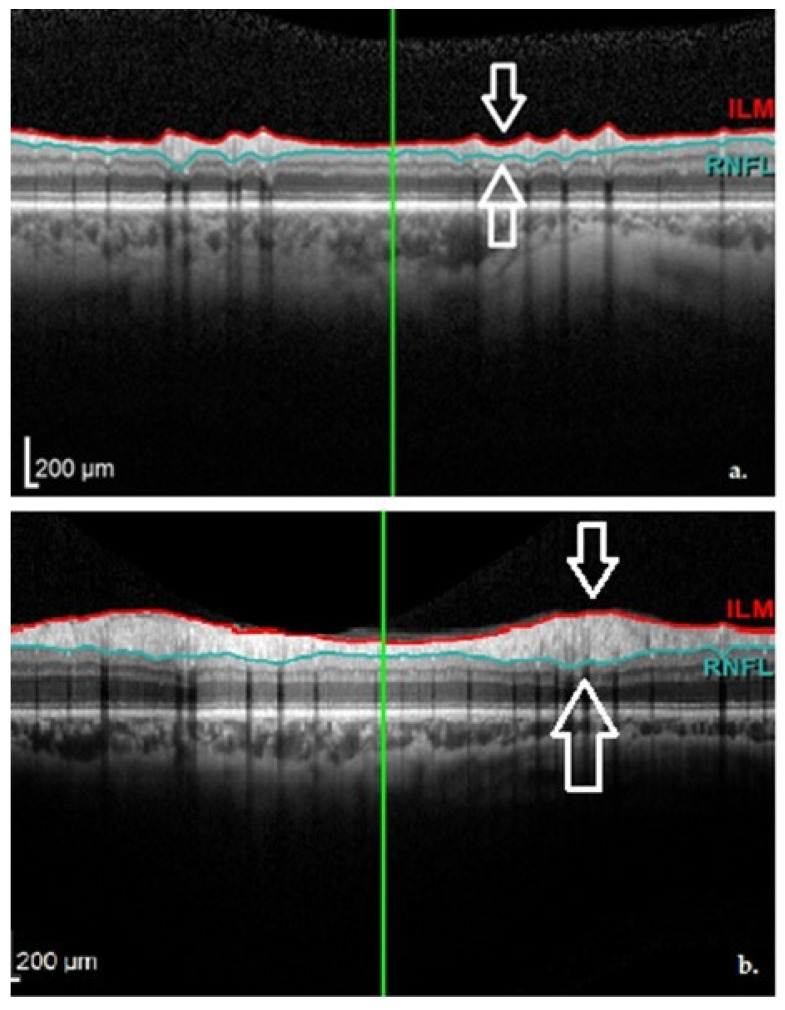
OCT aspect of the RNFL demonstrating thinning in a PD patient (**a**) and normal thickness in a healthy subject (**b**). Images from personal casuistry.

In 2014, Jimenez and colleagues studied the cpRNFL of 52 PD patients compared to healthy controls, and came up with a potential valuable formula that could predict disease severity. This was possible because of their observation that the cpRNFL gradually diminishes with the disease evolution. They also believed that OCT measurements could be useful tools in differentiating PD (see Figure 5) from other diseases, such as essential tremor [12]. This study, however, showed no correlation between the Hoehn−Yahr stage and the global cpRNFL value (µm) with *r* = 0.121 in the PD sample.

### 4.3. Lateralization of the Parkinsonism and cpRNFL Thinning

Our study reveals an important link to lateralization of the cpRNFL with parkinsonism, showing that the predominant retinal thinning measured by OCT was contralateral to the parkinsonism (*p* = 0.001). Previous studies examined randomly only one eye, but this method fails to take into consideration the possibility of lateralization of the parkinsonism regarding retinal thinning [13]. Lateralization can only be revealed when examining both eyes, as this study showed. This observation could further increase our knowledge of the circuitry and pathogenesis in PD.

Lateralization was also confirmed by a few other studies. cpRNFL thinning reflects the loss of the ganglion cells. Some authors concluded that dopaminergic loss can cause structural changes in the retina. It has also been found that foveal thickness (OCT) is reduced in the eye that is contralateral to the tremor and parkinsonism, both in PD and essential tremor patients. This reveals the asymmetry of these diseases [19].

On the other hand, from a clinical (practical) point of view, considering screening larger populations of PD patients for visual function changes could raise problems regarding evaluation times. Each thorough ophthalmologic examination (including OCT) of each patient takes time. This generates delays in examining large PD populations and reduces the chance for timely and optimal therapeutic management. This in turn decreases the QoL for our patients. A time-saving solution could represent scanning only one eye (the left retina) in the screening process, as we showed that the left retina was predominantly affected (*p* = 0.001) in our PD sample. Despite this, more data are still needed to confirm this supposition and to establish screening programs for visual function changes in PD patients.

In 2018, Adams et al. wrote an editorial about the future biomarkers for the diagnosis of PD and reaffirmed the potential value of the OCT in diagnosing PD in early stages. He also approached the link between the basal ganglia and the retina, demonstrated by studies showing an asymmetric thinning of the RNFL in the contralateral eye to the more affected body side. OCT is considered a biomarker for PD, along with clinical, biochemical, genetic, and digital ones [21].

### 4.4. Miscellaneous Observations and Differences between Measurements

Bodis-Wollner stressed that various OCT devices can lead to differences in measurements, and thus it is very important to compare the measurements on the same device. Because of the inter-ocular asymmetry, both eyes should be examined [20]. Taking into account these observations, in our study, we examined all subjects with the same OCT device and considered both eyes for the analysis. Furthermore, in order to eliminate the potential inter-examiner differences, all subjects were examined by the same neurologist and ophthalmologist.

The standard deviation between cpRNFL measurements was higher in some cases, probably due to a large dispersion of measurements between individuals. This could signify that some age groups had a larger spread of cpRNFL values among the subjects (inter-individual variability). With regard to this, for future studies, it would be beneficial to have a large sample size in order to eliminate such errors.

Some authors have found thinning of the inner retinal layers in the macular region. The fovea is considered to be important in color vision and contrast sensitivity because of the richness in photoreceptors. Therefore, foveal thinning should explain the reduced color discrimination and contrast sensitivity in PD patients. Garcia-Martin et al. found a negative correlation between the thickness of the nerve fiber layer (representing the axons of the ganglion cells of the retina) and the Hoehn−Yahr staging, explaining the possibility that foveal thickness may predict the severity of PD. Other authors have not found any correlations regarding this issue [17,20]. Our study, like others, found no correlation between the Hoehn−Yahr stage and the global cpRNFL value (µm) in the entire PD sample of all age groups. This can be explained by our finding, that retinal thinning is significant in PD patients of ages < 70 years in the earlier stages of evolution.

Despite the fact that 30 to 60% of PD patients develop visual hallucinations (VH) in the evolution of the disease, Kopal and colleagues believe that these symptoms have no link to the retinal changes, because they did not find any retinal thickness differences between 15 PD patients with VH compared to 15 PD patients without VH [30]. In the present study, there were no subjects complaining of VH, probably due to the inclusion of PD patients in the early stages of evolution. In addition, the lack of these complications could originate in the fact that all included PD subjects had optimal dopaminergic treatment and the MMSE scores were > 20 in all patients.

In 2018, in an editorial, Onofrj and Gilbert estimated the prevalence of the VH related to PD to be 22–38%. The VH seemed to be correlated with increased mortality and cognitive decline. They cited Firbank et al., who, with the help of Magnetic resonance spectroscopy, demonstrated that in PD with complex VH there were reduced concentrations of GABA in the visual cortex, thereby exonerating the retina for being implicated in the appearance of the complex VH [31].

In 2019, Leyland and associates studied the retinal volume in PD patients with dementia, and concluded that the thinning of the ganglion cell layer and the inner plexiform layer was correlated with the risk of PD dementia [32]. In this study, we showed that most PD subjects had MMSE scores of 25–29 points (50%) and 30 points (38.8%). In addition, the Hoehn−Yahr grading scale showed an approximate similar distribution between stages 2–3.

The gender distribution of the PD subjects in our study showed an asymmetry, favoring men (78%) versus women (22%). Despite this, according to Mauschitz et al. (2019), there were no significant differences between cpRNFL SD-OCT measurements between men and women (*p* = 0.42) in 1227 Caucasian individuals [33].

## 5. Conclusions

Nowadays, retinal OCT measurements have rapidly growing applications both in ophthalmology and in neurology. Because the retina is the gateway towards examining the brain in-vivo, we tried to use this valuable structure in order to investigate possible changes in PD patients.

Our research confirms the results of recent studies, revealing specific patterns of cpRNFL thinning in PD patients. OCT has demonstrated its usefulness in other neurological disorders such as multiple sclerosis. It has also been previously speculated that OCT could be a potential candidate for generating a valuable marker for diagnosing PD. The reliability of its measurements is still under debate; therefore, publishing personal results is useful for outlining the status of OCT in PD.

To the best of our knowledge, this is the first dedicated study regarding the visual function changes in PD to be done in a Clinical Research Hospital in the Transylvania Region, Romania, reporting the results of the cpRNFL SD-OCT imaging in PD patients. Our results could be a starting point for implementing OCT in the examination protocol of patients with PD, and could possibly lead us towards reaching more personalized medicine.

The strengths of our study are as follows: it is the first study performed in a Clinical Research Hospital in the Transylvania region, Romania, confirming the other research observations regarding the cpRNFL measurements in PD; it brings new information about the distinctive cpRNFL changes correlated with age; it brings new evidence supporting the theory of retinal changes’ lateralization with respect to parkinsonism; and it strengthens the connection between the morphological retinal changes revealed by OCT and the ophthalmological chart evaluations.

Our study is limited by the relatively small sample size, as it was done in one hospital. Therefore, further research is mandatory to confirm the present data. Future studies could address the issue of diagnosing PD in early or prodromal stages with the help of a multimodal approach, using multiple imaging devices (OCT and fMRI). The practical outcome of the study is represented by the need of continuous ophthalmological monitoring of PD patients in order to identify further changes along the evolution of this condition over time.

## Figures and Tables

**Table 1:** Distribution of the PD sample according to the clinical subtypes.

Gender	Equivalent PD Type	Akinetic-Rigid Predominant PD Type	Tremor Predominant PD Type
Women %	0	5.5	16.5
Men %	6	22	50

**Table 2:** Lateralization for each PD patient studied.

	Parkinsonism Predominant Side	Predominant Retinal Layer Thinning
PD Patient 1	2	1
PD Patient 2	2	1
PD Patient 3	2	1
PD Patient 4	2	1
PD Patient 5	2	0
PD Patient 6	2	1
PD Patient 7	2	1
PD Patient 8	2	1
PD Patient 9	2	2
PD Patient 10	2	0
PD Patient 11	2	1
PD Patient 12	1	1
PD Patient 13	1	1
PD Patient 14	1	2
PD Patient 15	1	1
PD Patient 16	1	2
PD Patient 17	1	1

Notes: 0—equivalent; 1—left side; 2—right side.

**Table 3:** Retinal layer thickness in PD patients and controls.

**40–49 Years–Age Group**					
**cpRNFL Values (μm)**	**PD Mean ± SD**	**HC Mean ± SD**	**ANOVA *p* Value**	***t*-Test *p* Value**	**Bonferroni Method** **Correction** **Significance**
Temporal	63 ± 4.24	87 ± 11.39	0.05	0.005	Yes
TS	132 ± 8.48	153.67 ± 21.68	0.244	0.051	No
NS	93.5 ± 3.53	127.33 ± 19.72	0.03	0.001	Yes
N	82 ± 0	87.67 ± 8.48	0.55	0.150	No
NI	92 ± 11.31	119.67 ± 12.64	0.035	0.053	No
TI	142.5 ± 6.36	171.67 ± 21.1	0.203	0.029	No
Global	93 ± 2.12	112 ± 7.38	0.02	0.001	Yes
**50–59 Years–Age Group**					
**cpRNFL Values (μm)**	**PD Mean ± SD**	**HC Mean ± SD**	**ANOVA *p* Value**	***t*-Test *p* Value**	**Bonferroni Method** **Correction** **Significance**
Temporal	53 ± 7.07	91 ± 5.65	0.027	0.015	Yes
TS	107 ± 7.07	150.50 ± 12.02	0.047	0.034	No
NS	130 ± 4.24	139 ± 5.65	0.213	0.111	No
N	73 ± 7.07	67.5 ± 0.7	0.387	0.234	No
NI	106 ± 4.24	85.5 ± 4.94	0.047	0.024	Yes
TI	115.5 ± 4.94	144.5 ± 10.6	0.072	0.058	No
Global	88.5 ± 0.7	101 ± 0	0.001	0.012	Yes
**60–69 Years–Age Group**					
**cpRNFL Values (μm)**	**PD Mean ± SD**	**HC Mean ± SD**	**ANOVA *p* Value**	***t*-Test *p* Value**	**Bonferroni Method** **Correction** **Significance**
Temporal	63.7 ± 10.78	75.73 ± 9.613	0.014	0.007	Yes
TS	123.3 ± 25.04	142.18 ± 14.15	0.044	0.027	No
NS	105.8 ± 17.2	113.73 ± 21.91	0.371	0.183	No
N	72.3 ± 18.64	82.64 ± 7.87	0.108	0.065	No
NI	105.3 ± 15.04	120.27 ± 15.74	0.038	0.019	Yes
TI	123.3 ± 26.11	165.27 ± 15.45	0.0002	0.0002	Yes
Global	88.4 ± 17.83	104.36 ± 4.88	0.01	0.010	Yes
**70–79 Years–Age Group**					
**cpRNFL Values (μm)**	**PD Mean ± SD**	**HC Mean ± SD**	**ANOVA *p* Value**	***t*-Test *p* Value**	**Bonferroni Method** **Correction** **Significance**
Temporal	65.57 ± 12.53	72 ± 2.82	0.493	0.068	No
TS	128.35 ± 31.31	136.5 ± 12.02	0.727	0.268	No
NS	118.35 ± 19.99	120.5 ± 0.7	0.885	0.348	No
N	80.57 ± 16.91	87 ± 2.82	0.61	0.108	No
NI	124.42 ± 26.64	122 ± 5.65	0.902	0.386	No
TI	136.21 ± 14.66	147 ± 12.72	0.342	0.212	No
Global	99.78 ± 13.07	103 ± 1.41	0.74	0.195	No

cpRNFL—circumpapillary retinal nerve fiber layer; PD—Parkinson’s disease; HC—healthy controls; TS—temporal-superior quadrant; NS—nasal-superior quadrant; N—nasal quadrant; NI—nasal-inferior quadrant; TI—temporal-inferior quadrant.

**Table 4:** Regression summary.

	Dependent Variable	R Square	Significance F	Visual Symptoms (*p* Value)	Non-Motor Symptoms (*p* Value)	Amsler Grid (*p* Value)	Ishihara Test (*p* Value)	Pelli-Robson (*p* Value)
Right eye	Temporal	67%	0.017	0.136	0.061	0.065	0.014	0.928
	NI	47%	0.155	0.694	0.066	0.486	0.151	0.541
	Global	67%	0.016	0.787	0.040	0.550	0.004	0.242
Left eye	Temporal	60%	0.045	0.293	0.011	0.874	0.047	0.728
	NI	48%	0.139	0.261	0.186	0.405	0.256	0.443
	Global	45%	0.183	0.395	0.048	0.971	0.094	0.592

## Data Availability

Patient data were anonymized. The data are not publicly available due to restrictions (ethical and privacy).
